# Design of optimal Elman Recurrent Neural Network based prediction approach for biofuel production

**DOI:** 10.1038/s41598-023-34764-x

**Published:** 2023-05-26

**Authors:** N. Paramesh Kumar, S. Vijayabaskar, L. Murali, Krishnaraj Ramaswamy

**Affiliations:** 1 Department of Electrical and Electronics Engineering, P.A. College of Engineering and Technology, Pollachi, India; 2 Department of Electronics and Communication Engineering, P.A. College of Engineering and Technology, Pollachi, India; 3 Centre for Excellence-Indigenous Knowledge, Innovative Technology Transfer and Entrepreneurship, Dambi Dollo University, Dambi Dollo, Ethiopia; 4 Department of Mechanical Engineering, Dambi Dollo University, Dambi Dollo, Ethiopia

**Keywords:** Chemistry, Energy science and technology, Engineering, Mathematics and computing

## Abstract

Renewable sources like biofuels have gained significant attention to meet the rising demands of energy supply. Biofuels find useful in several domains of energy generation such as electricity, power, or transportation. Due to the environmental benefits of biofuel, it has gained significant attention in the automotive fuel market. Since the handiness of biofuels become essential, effective models are required to handle and predict the biofuel production in realtime. Deep learning techniques have become a significant technique to model and optimize bioprocesses. In this view, this study designs a new optimal Elman Recurrent Neural Network (OERNN) based prediction model for biofuel prediction, called OERNN-BPP. The OERNN-BPP technique pre-processes the raw data by the use of empirical mode decomposition and fine to coarse reconstruction model. In addition, ERNN model is applied to predict the productivity of biofuel. In order to improve the predictive performance of the ERNN model, a hyperparameter optimization process takes place using political optimizer (PO). The PO is used to optimally select the hyper parameters of the ERNN such as learning rate, batch size, momentum, and weight decay. On the benchmark dataset, a sizable number of simulations are run, and the outcomes are examined from several angles. The simulation results demonstrated the suggested model's advantage over more current methods for estimating the output of biofuels.

## Introduction

The global energy system is based largely on fossil fuels^1^. The significance of energy system also its part in politics and economics isn’t hidden for everyone. This problem is significant for innovative industrialized countries that are the main energy consumer, as well as significant for oil-rich countries^2^. Since countries must comprehend the fact that fossil fuels resource is constrained resource. Besides the nature of this fuel have polluting substance, the problem oftheir ended up has provoked the increased attention. Due to the fact that reducing pollution, environmental damage, and non-renewable energy resources the world is turning toward sustainable energy resources^3^. Still, fossil fuel remains the main energy resource around the world. Highly based fossil fuel has created an energy crisis. Utilizing fossil fuels for economic activity lead to greenhouse gas (GHG) emissions from most region of the world. Because of the environmental concern and the fluctuation and rise in the fossil fuels resource, global interest has movedtoward biodiesels, renewable and clean alternatives for fossil fuel^4^. Biofuel is employed indistinct areas for producing energy such as transportation, power, and electricity production.

The importance of energy systems and their role in economics and politics is not hidden for anyone. This issue is not only important for the advanced industrialized countries, which are major energy consumers but is also essential for oil-rich countries. In addition to the nature of these fuels, which contains polluting substances, the issue of their ending up has aggravated the growing concern. Biofuels can be used in different fields for energy production like electricity production, power production, or for transportation^5^. Various scenarios have been written about the estimated biofuels from different sources in the future energy system. The availability of biofuels for the electricity market, heating, and liquid fuels is critical. Accordingly, the need for handling, modeling, decision making, and forecasting for biofuels can be of utmost importance.

Statistical and Mathematical model provides basic data to understand, analyze, and predict biological process, and are essential for optimizing significant parameter for improving system efficiency^6^. Optimization and Modeling of biofuels manufacturing process would participate to understand better of the expenditure procedure for obtaining an optimal efficacy. The primary objective of modelling is to enhance the operation involving in their productions for achieving efficacy development. Artificial intelligence tools had emerged as possible methods to optimize and model bioprocess. Over the previous years, artificial neural network (ANN) was used in nonlinear, multidimensional development and research of bioprocess. They have proved their effectiveness in emerging bioprocesses model lacking of previous data on them etabolic and kinetics flow occurs in cell and cells surrounding^7^.

Moreover, ANN is dependent fully on data, with no previous experience on the event regulate the procedure^8^. The appeal of ANN as modelling tools derive from their exclusive function of processing data i.e., allocated high parallelism, primarily—linearity, and noise and error acceptance—and their ability to generalize and learn. ANN has received more interest from substantial soft computing tool which is constrained only for data analysis and processing, however, could also be employed for solving problem in nonlinear and multifaceted procedures^9^.

In recent times, deep learning and machine learning methods have been widespread in handling, modeling and optimizing the biodiesel consumption, production and its environmental impact by taking into account the effects of parameter on biofuel yields since production of a preferred products need an efficient usage of investigational models. This method provides a self-governing modeling method to the nature of procedure or its arithmetical model as well as capable of modelling the procedure using higher performances^10^.

In this research, a new optimal Elman Recurrent Neural Network (OERNN) based prediction model for biofuel prediction is proposed which provides better result when compared with the other existing approaches. The OERNN-BPP technique involves empirical mode decomposition (EMD) based pre-processing and fine to coarse (FTC) based reconstruction model. Besides, ERNN model is employed for the prediction of biofuel productivity. For enhancing the predictive performance of the ERNN model, a hyper parameter optimization process takes place using political optimizer (PO). A comprehensive experimental analysis is carried out on benchmark dataset and the results are examined interms of diverse aspects.

### Related works

This section provides numerous research studies that have been focussed based on the production of biofuel. To gain a better understanding of the literal works and the relevant research areas are summarized as follows. The production of biofuel based on spatial distribution was implemented by Elmore et al.^11^ where this approach utilized the Moderate Resolution Imaging Spectrometer (MODIS). Here, the residue from the rice was employed to produce the biofuel. The accuracy and the flexibility rate were very high in this approach; on the other hand, there occurs a complexity in designing the spatial model.

Chanthawong et al.^12^ proposed two different types of approaches namely two stages least square and three stages least square for biofuel production in the Thailand market. This dual-stage approach is developed with minimum cost with very less dynamic model constitution. The accurate biogas prediction was developed by De et al.^13^ that utilized the neural network model namely k-nearest neighbors (KNN) for the effective production of biofuel. Here the forecasting accuracy is very high with improved facility performances. But, there occur a few complexity issues during implementation. Then Dehghani et al.^14^ demonstrated a future forecasting model based on the production of biofuel and this approach utilized seven biofuel technologies namely gas turbine, Combined Heat and Power (CHP) turbine, bio-pyrolysis, cellulosic bioethanol, grain bioethanol, torrefaction and biodiesel. Moreover, the execution performance and the accuracy were very high; but the research and development of this approach are not much efficient.

Radivojević et al.^12^ introduced the Automated Recommendation Tool (ART), a tool which leverage ML and probabilistic approaches for guiding synthetic biology from the systematic fashion, with no requirement to complete mechanistic kind of biological scheme. The following engineering cycle's group of recommended strains, along with a probabilistic prediction of its production level, are provided by ART using sampling-based optimization.

Elveny et al.^13^ presented a novel Machine Learning (ML) technique dependent upon Extreme Learning Machine (ELM) for modelling this essential value. The real database involving 483 actual datasets has been related to the output forecasted with ELM technique. In Cui et al.^14^, distinct ML techniques are estimated to be the primary time for establishing the forecast technique amongst biodiesel composition and cold filter plugging point (CFPP). The decision trees (DT) based techniques are optimum efficient in forecasting CFPP of biodiesel.

Kumar et al.^15^ aimed in evolving a new Adaptive Integrated Optimization Network (AION) for attaining optimum biofuel production with maximal accuracy as well as minimal error value rates. Also, the presented AION manner includes 4 important stages as Pre-processing of data, Re-construction of components, Prediction of individuals, and Ensemble predicting. Javed et al.^16^ developed a grey predicting technique with optimized the model frame work (data accumulation function and background value generation). The presented predicting technique, Even form of Grey Forecasting model (EGM) (1,1,α,θ) is a generalization procedure of the even procedure of grey predicting technique and their comparative efficiency turned out that commonly higher than that of the original technique.

Beeravalli et al.^17^ search a new manner for classifying feed stock’s utilizing secondary works data sources. Also, the maximum reliability of techniques utilized, the study analyzed investigating over 20 parameters of 106 feed stocks. The study established a rating scheme to Multi-Criteria Decision Analysis (MCDA) containing weighting to all parameters dependent upon expert opinion or statistical techniques namely Principal Component Analysis (PCA). The ranking method output afterward is fed as to Multivariate Regression (MVR) and Multilayer perceptron (MLP), for ranking feed stocks for producing the maximum quality maintainable biofuels to a specific place.

Geng et al.^18^ resolved the influence of random fluctuation data and weak anti-interference capability in the Markov chain model by proposing a dynamic fuzzy grey-Markov prediction model for biofuel production forecasting, in order to improve the prediction performance of the conventional prediction methods based upon past production levels in conjunction with the factors of economy, governmental policies, and technological developments. Their empirical results demonstrated the superiority of the proposed fuzzy grey-Markov model relative to the benchmark prediction models. However, the biofuel production system is a complex system, which is affected easily by various factors such as the economy, governmental policies, resources, technological developments and social issues. Thus, the above methods can only provide good prediction results under linear assumptions, being unable to capture the hidden nonlinear features of the biofuel production series. Numerous experiments have demonstrated that the predictive performance may be very poor if one uses these traditional statistical and econometric methods (Mejdoub and Ghorbel^19^; Song and Yu^20^; Weigend^21^). Therefore, the traditional methods are not suitable for predicting biofuel production (Geng et al.^18^).

### The proposed model

In this study, a new OERNN-BPP technique has been presented to predict the productivity of biofuels. The OERNN-BPP technique follows four major processes namely EMD based processing, FTC based reconstruction, ERNN based prediction, and PO based hyper parameter optimization. The design of PO based hyper parameter tuning process assists in optimally adjusting the learning rate, batch size, momentum, and weight decay.

**Figure 1:**
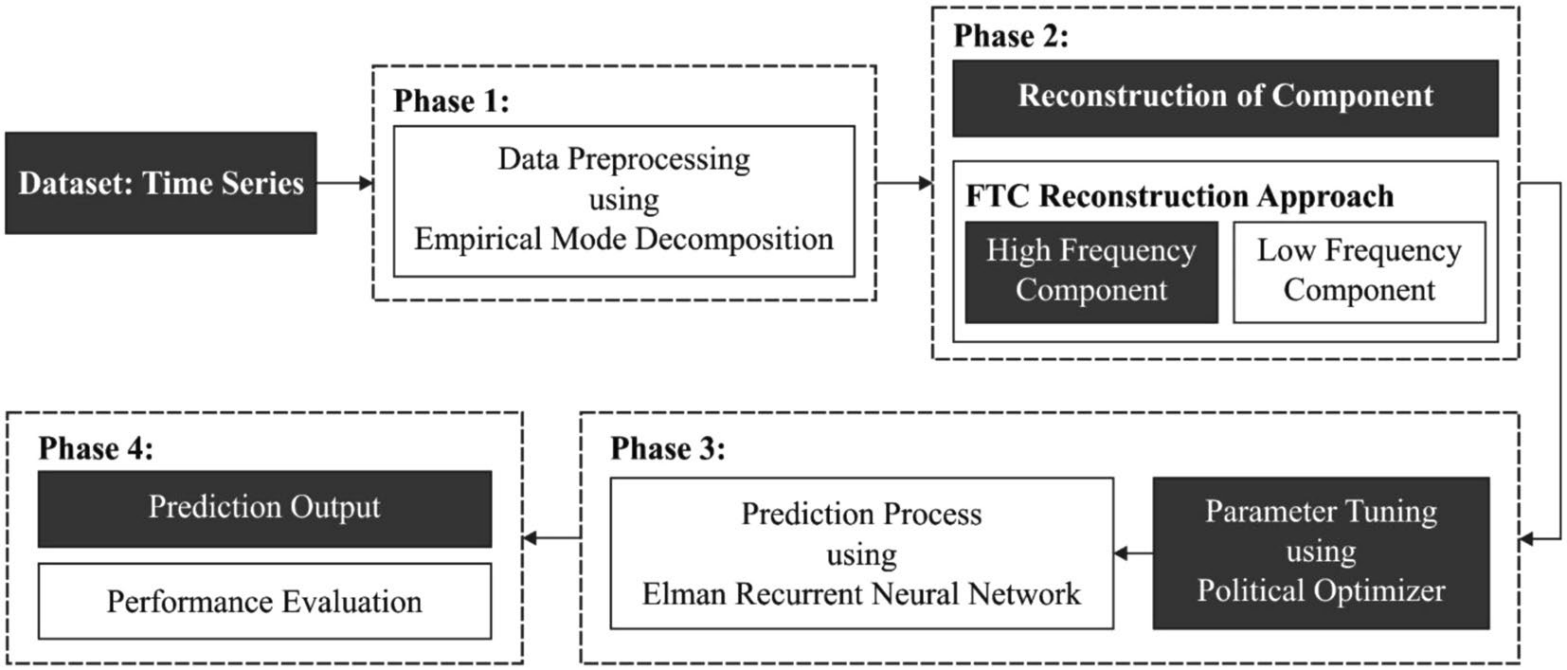
Overall block diagram of OERNN-BPP model.

Figure 1 demonstrates the overall block diagram of OERNN-BPP model. The detailed working of these processes is discussed in the succeeding sections.

### EMD based data pre-processing

Initially, a decomposition method called EMD is used in separating raw complex data into relatively simple/uncomplicated data thus decreasing the complication problems. Next, the EMD method is related to other decomposition methods like the Fourier decomposition and wavelet decomposition approaches^22^. Now, the EMD method, i.e., a type of intuitive, self-adapting, empirical and direct method and is suited well for nonlinear and non-stationary data. Generally, the EMD method employed in decomposing the raw time sequence data to some periodic mode functions *I*_*MF*_ contain independent data. Accurately, the IPMF fulfills 2 distinct criteria’s which are given below.

1 $$Criteria:\left\{\begin{array}{cc}{N}_{E}\, and\, {N}_{ZC}\, are\, equal\,; & \forall \, Each\, whole\, funct\,\left(i.e. \mathrm{0,2},4\dots \right)\\ {A}_{T }\, \left(T=\mathrm{1,2},3,\dots \right); & \forall \,Symmetric\, function\end{array}\right.$$

From Eq.(1), *N*_*E*_* & N*_*ZC*_ denotes the overall amount of extrema and zero crossing correspondingly. The novel data depends on time sequence is given by A_T_; whereas its expressions are signified as follows.

2 $${A}_{T}=\sum\limits_{I=1}^{M}{B}_{I,}+{R}_{M,}$$

From Eq. (2), the overall quantity of *I*_*MF*_ and there s i due at a time ‘*T*’ is denoted by M & *R*_*M*,_ correspondingly.

### FTC based reconstruction

The FTC model reconstructs IMF as 2t-testing parts namely minimum and maximum frequency elements. Inaddition, thet-testing frequency element comprises distinct features involving the information about the centralized traits. So, a simpler structure of FTC model is applied for improving the accuracy and reducing the computational complexity. The FTC model encompasses a 2-stage process. Firstly, the preprocessed IMF attained from the earlier level gets inspected by the use of t-testing. The next level includes the choice of IMF similarity including unrelated divergences at a certain degree of confidence are reconstructed different elements. The classification of maximal and minimal frequency elements is then done using the IMF with closer IMF over t-testing if the IMF resemblance with irrelevant divergence at a certain level of confidence cannot be achieved.

### Design of ERNN based predictive model

**Figure 2:**
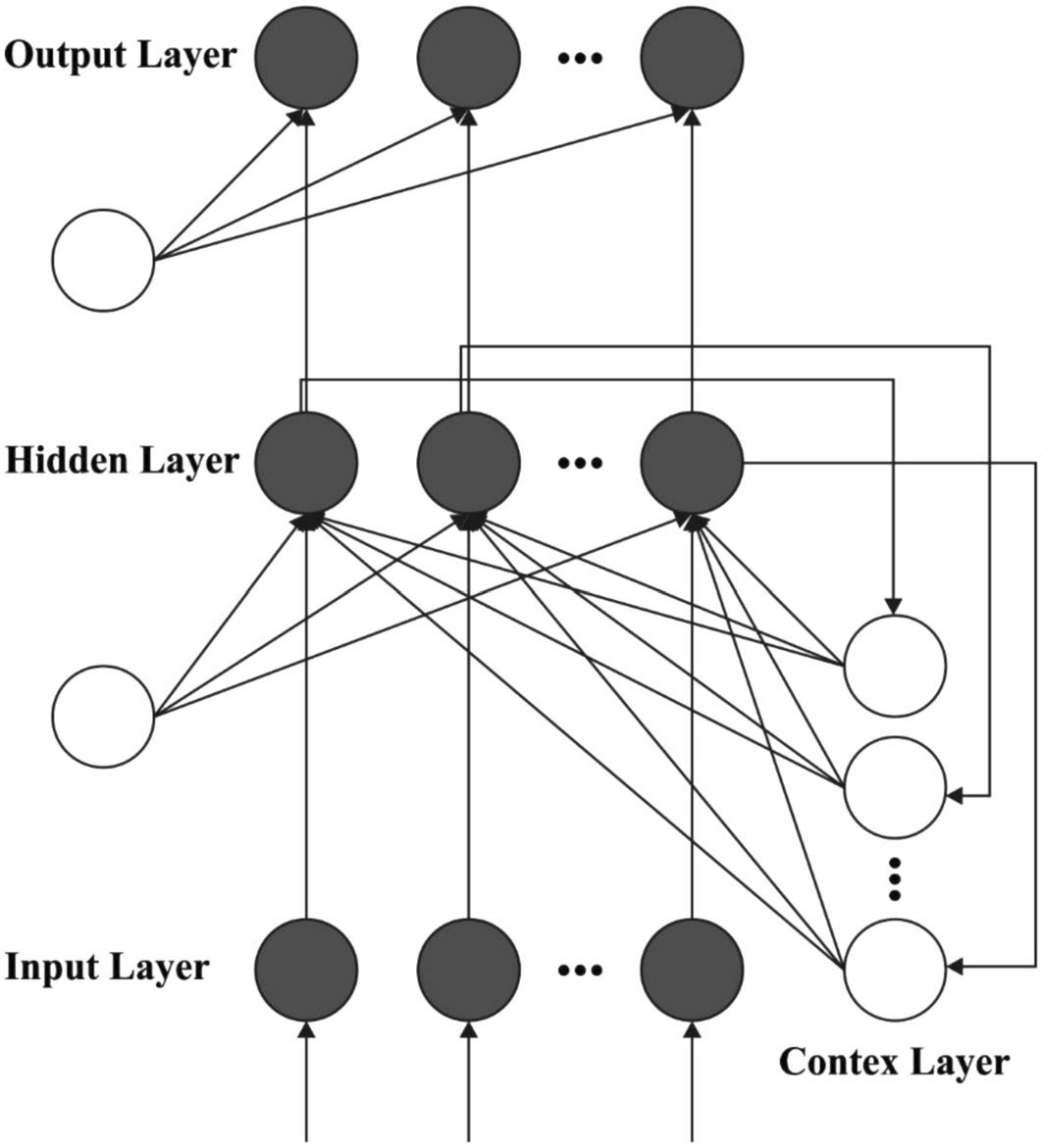
Structure of ERNN.

At this stage, the ERNN model receives the input and predicts the actual production of biofuels. The ERNN has been simple RNN is established by Elman in 1990^23^. Already known, are current network is a few benefits like consuming time series and non-linear forecast abilities, faster convergence, and further accurate mapping capability. They combine Elman neuralnetwork (ENN) with distinct regions to its purpose. During this network, the outcomes of the hidden layers (HL) were permitted to feedback on itself with butter layer is named as recurrent layers (RL). Figure 2 illustrates the architecture of ERNN model.

This feedback enables ERNN to learn, recognise, and produce spatial patterns as well as temporal designs. One RL neuron with a constant weight of one connects all hidden neurons. As a result, the RL almost creates a copy of the HL's previous instantaneous state. Accordingly, the number of recurrent neurons is comparable to the number of hidden neurons. Each layer has several neurons that propagate information from one layer to the next by computing a nonlinear function of the inputs' weighted sum.

The multi‐input ERNN technique has been demonstrated, in which the amount of neurons from the input layers are *m* and during the HL is *n* and one output unit. Assume that *x*_*it*_(*i* = 1,2, … , *m*) represents the group of input vector of neurons at time *t*, *y*_*t*+1_ implies the outcome of networks at time *t* + 1, *z*_*jt*_ (*j* = 1,2,…,*n*) refers the outcome of HL neuron at time *t*, and (*j* = 1,2, …, *n*) defines the RL neuron. *w*_*ij*_ signifies the weight that links the node *i* from the input layer neuron to node *j* from the HL. *c*_*j*_, are the weights which link the node *j* during the HL neurons to node under the RL and output correspondingly. The HL stage is as follows: the inputs of every neuron from the HLs are provided as:

3 $$\begin{aligned} \left( {\text{k}} \right) = & \mathop \sum \limits_{{{\text{i}} = 1}}^{{\text{n}}} {\text{w}}_{{{\text{ij}}}} {\text{x}}\left( {{\text{k}} - 1} \right) + \mathop \sum \limits_{{{\text{j}} = 1}}^{{\text{m}}} {\text{c}}_{{\text{j}}} {\text{u}}_{{{\text{jt}}}} \left( {\text{k}} \right) \\ {\text{u}}_{{{\text{jt}}}} \left( {\text{k}} \right) = & {\text{z}}_{{{\text{jt}}}} \left( {{\text{k}} - 1} \right),\; {\text{i}} = 1,2, \ldots ,{\text{n}}, \;{\text{j}} = 1,2, \ldots ,{\text{m}}. \\ \end{aligned}$$

The outcomes of hidden neurons are provided as:

4 $${z}_{jt}\left(k\right)={f}_{H}\left({net}_{jt}\left(k\right)\right)=\left(\sum\limits_{i=1}^{n}{w}_{ij}\left(k\right)+\sum\limits_{j=1}^{m}{c}_{j}{u}_{j}\left(k\right)\right)$$

where the sigmoid function from HL has been elected as activation function: *f*_*H*_(*x*) = 1/(1 + *e*^−χ^). The outcome of HL is provided as^24^:

5 $${y}_{t+1}\left(k\right)={f}_{T}\left(\sum\limits_{j=1}{v}_{j}\left(k\right)\right)$$

where (*x*) refers the identity map as activation functions.

### Design of PO based hyper parameter tuning process

For enhancing the predictive outcome of the ERNN model, the hyperparameter tuning process is carried out using PO. PO is a recently developed Meta heuristic approach that depends on human behaviour and is stimulated from the multiphase PO. But, it has to be mentioned that the presented method isn’t primary of these kinds. In PO, the concepts of politics are mappedfrom a distinct point of view and different from the current politics stimulated algorithm, and it is because of the 4 reasons. Initially, PO tries to model every major step in politics like party development, constituency distribution or party ticket, party transferring, election campaign, parliamentary affairs, and interparty election afterward governments creation.

Next, PO presents a new location upgrading approach named RPPUS. This later represents the learning performance of the politician from the former election. Then, all the individuals’solutions assume a binary task: an election candidate and party member. With these concepts, every solution could be upgraded based on the 2 optimal solutions: constituency winner and party leader. Lastly, in order to enhance the result, intermediary solutions need to communicate and cooperate through a phase called parliamentary affairs. In PO, all the party members are regarded as a candidate solution in which its good will has deliberated the location in the search spaces. Furthermore, the calculation functions are processed in the course of the election stage whereas the numbers of votes attained by all the party members represent the fitness of candidate solutions. PO model is generated using the 5 major stages in the following: constituency allocation, party development, party transferring, election campaign, parliamentary affair, and interparty election^25^. It has to be noted that the initial stage (constituency allocation and party formation) is performed once for initializing and affects distinct parameters.

#### Party formation and constituency allocation

At first, the population *P* is divided into *N* party, whereas all the parties *P*_*i*_ include *N* member (possible solutions). Furthermore, all *jt*ℎ members are referred to as *P*^*j*^ and denoted as a *d* dimension vectors, in which the values *d* are the amount of input parameters of the processed problems and ${P}_{i,k}^{j}$ denote kth dimensions of As above-mentioned, all the members have deliberated as an election candidate as well itsrole as a member party. Therefore, *N* constituency is made and have *jt*ℎ members of all contesting parties. Moreover, the leader of the ith party afterward calculating the fitness of each member is stated as ${P}_{i}^{*}$ and the group of each party leader is given as *P*^∗^. Incontrast, afterward the election, *C*^∗^ regroup the winner from each constituency called the parliamentarian, whereas ${C}_{j}^{*}$ denote the winners of jth constituencies.

#### Election campaign

In this stage, party member is trying to improve their chance of being selected by altering their position based on the 3 factors. Firstly, they attempt for learning from prior knowledge with a new location upgrading approach named RPPUS as expressed in Eqs. (6) & (7). Next, all-party members are trying to upgrade their present location based on the party leader. Lastly, candidate position is updated regarding the constituency winners:

6 $$\begin{array}{l} {\rm{P}}_{{\rm{i}},{\rm{k}}}^{\rm{j}}\left( {{\rm{t}} + 1} \right) = \\ \begin{array}{*{20}{l}} {{{\rm{m}}^*} + {\rm{r}}\left( {{{\rm{m}}^*} - {\rm{P}}_{{\rm{i}},{\rm{k}}}^{\rm{j}}\left( {\rm{t}} \right)} \right),}&{{\rm{if}}\;{\rm{P}}_{{\rm{i}},{\rm{k}}}^{\rm{j}}\left( {{\rm{t}} - 1} \right) \le {\rm{P}}_{{\rm{i}},{\rm{k}}}^{\rm{j}}\left( {\rm{t}} \right) \le {{\rm{m}}^*}\quad {\rm{or}}\;{\rm{P}}_{{\rm{i}},{\rm{k}}}^{\rm{j}}\left( {{\rm{t}} - 1} \right) \ge {\rm{P}}_{{\rm{i}},{\rm{k}}}^{\rm{j}}\left( {\rm{t}} \right) \ge {{\rm{m}}^*}}\\ {{{\rm{m}}^*} + \left( {2{\rm{r}} - 1} \right)\left| {{{\rm{m}}^*} - {\rm{P}}_{{\rm{i}},{\rm{k}}}^{\rm{j}}\left( {\rm{t}} \right)} \right|,}&{{\rm{if}}\;{\rm{P}}_{{\rm{i}},{\rm{k}}}^{\rm{j}}\left( {{\rm{t}} - 1} \right) \le {{\rm{m}}^*} \le {\rm{P}}_{{\rm{i}},{\rm{k}}}^{\rm{j}}\left( {\rm{t}} \right)\quad {\rm{or}}\;{\rm{P}}_{{\rm{i}},{\rm{k}}}^{\rm{j}}\left( {{\rm{t}} - 1} \right) \ge {{\rm{m}}^*} \ge {\rm{P}}_{{\rm{i}},{\rm{k}}}^{\rm{j}}\left( {\rm{t}} \right)}\\ {{{\rm{m}}^*} + \left( {2{\rm{r}} - 1} \right)\left| {{{\rm{m}}^*} - {\rm{P}}_{{\rm{i}},{\rm{k}}}^{\rm{j}}\left( {\rm{t}} \right)} \right|,}&{{\rm{if}}\;{{\rm{m}}^*} \le {\rm{P}}_{{\rm{i}},{\rm{k}}}^{\rm{j}}\left( {{\rm{t}} - 1} \right) \le {\rm{P}}_{{\rm{i}},{\rm{k}}}^{\rm{j}}\left( {\rm{t}} \right)\quad {\rm{or}}\;{{\rm{m}}^*} \ge {\rm{P}}_{{\rm{i}},{\rm{k}}}^{\rm{j}}\left( {{\rm{t}} - 1} \right) \ge {\rm{P}}_{{\rm{i}},{\rm{k}}}^{\rm{j}}\left( {\rm{t}} \right)} \end{array} \end{array}$$

7 $$\begin{gathered} {\text{P}}_{{{\text{i}},{\text{k}}}}^{{\text{j}}} \left( {{\text{t}} + 1} \right) = \hfill \\ \begin{array}{*{20}l} {{\text{m}}^{*} + \left( {2{\text{r}} - 1} \right)\left| {{\text{m}}^{*} - {\text{P}}_{{{\text{i}},{\text{k}}}}^{{\text{j}}} \left( {\text{t}} \right)} \right|,} \hfill & {{\text{if}}\;{\text{P}}_{{{\text{i}},{\text{k}}}}^{{\text{j}}} \left( {t - 1} \right) \le {\text{P}}_{{{\text{i}},{\text{k}}}}^{{\text{j}}} \left( {\text{t}} \right) \le {\text{m}}^{*} \quad {\text{or P}}_{{{\text{i}},{\text{k}}}}^{{\text{j}}} \left( {{\text{t}} - 1} \right) \ge {\text{P}}_{{{\text{i}},{\text{k}}}}^{{\text{j}}} \left( {\text{t}} \right) \ge m^{*} } \hfill \\ {{\text{P}}_{{{\text{i}},{\text{k}}}}^{{\text{j}}} \left( {{\text{t}} - 1} \right) + {\text{r}}\left( {{\text{P}}_{{{\text{i}},{\text{k}}}}^{{\text{j}}} \left( {\text{t}} \right) - {\text{P}}_{{{\text{i}},{\text{k}}}}^{{\text{j}}} \left( {{\text{t}} - 1} \right)} \right),} \hfill & {{\text{if}}\;{\text{P}}_{{{\text{i}},{\text{k}}}}^{{\text{j}}} \left( {t - 1} \right) \le {\text{m}}^{*} \le {\text{P}}_{{{\text{i}},{\text{k}}}}^{{\text{j}}} \left( {\text{t}} \right)\quad {\text{or P}}_{{{\text{i}},{\text{k}}}}^{{\text{j}}} \left( {{\text{t}} - 1} \right) \ge {\text{m}}^{*} \ge {\text{P}}_{{{\text{i}},{\text{k}}}}^{{\text{j}}} \left( {\text{t}} \right)} \hfill \\ {{\text{m}}^{*} + \left( {2{\text{r}} - 1} \right)\left| {{\text{m}}^{*} - {\text{P}}_{{{\text{i}},{\text{k}}}}^{{\text{j}}} \left( {{\text{t}} - 1} \right)} \right|,} \hfill & {{\text{if}}\;{\text{m}}^{*} \le {\text{P}}_{{{\text{i}},{\text{k}}}}^{{\text{j}}} \left( {{\text{t}} - 1} \right) \le {\text{P}}_{{{\text{i}},{\text{k}}}}^{{\text{j}}} \left( {\text{t}} \right)\quad {\text{or m}}^{*} \ge {\text{P}}_{{{\text{i}},{\text{k}}}}^{{\text{j}}} \left( {{\text{t}} - 1} \right) \ge P_{{{\text{i}},{\text{k}}}}^{{\text{j}}} \left( {\text{t}} \right)} \hfill \\ \end{array} \hfill \\ \end{gathered}$$

To balance among exploitation and exploration, a stage named party switching is initiated afterward the election campaign stage. With adaptive parameters, called party switching rate, all party members *P*^*j*^ could be elected and switch to few arbitrarily selected party *P*_*r*_. Henceforth, it is exchanged with the minimum fit party member *P*_*r*_.

#### Election

This stage's aim is to calculate the fitness of each candidate contest in constituency. Afterward, the party leader and constituency winner are upgraded by:

8 $$\begin{aligned} {\text{q}} = &\, {\text{argmin}}\left( {{\text{P}}_{{\text{l}}}^{{\text{j}}} } \right),1 \le {\text{i}} \le {\text{N}}, \\ {\text{C}}_{j}^{*} = &\, {\text{P}}_{{\text{q}}}^{{\text{i}}} , \\ {\text{P}}_{{\text{j}}}^{*} = & \,{\text{P}}_{{\text{q}}}^{{\text{i}}} \\ \end{aligned}$$

### Parliamentary affairs

Afterward defining the party leader and constituency winner (parliamentarian), all the parliamentarians aiming to enhance their performances by mimicking the cooperation and interaction of the winning candidate to manage the governments in the post-election stage. All the parliamentarians *C*_j_^∗^ update its location regarding arbitrarily selected parliamentarians *C*_r_^∗^. It has to be pointed out that the movements are used only when the performances of*C*_r_^∗^ are improved.

**Figure 3:**
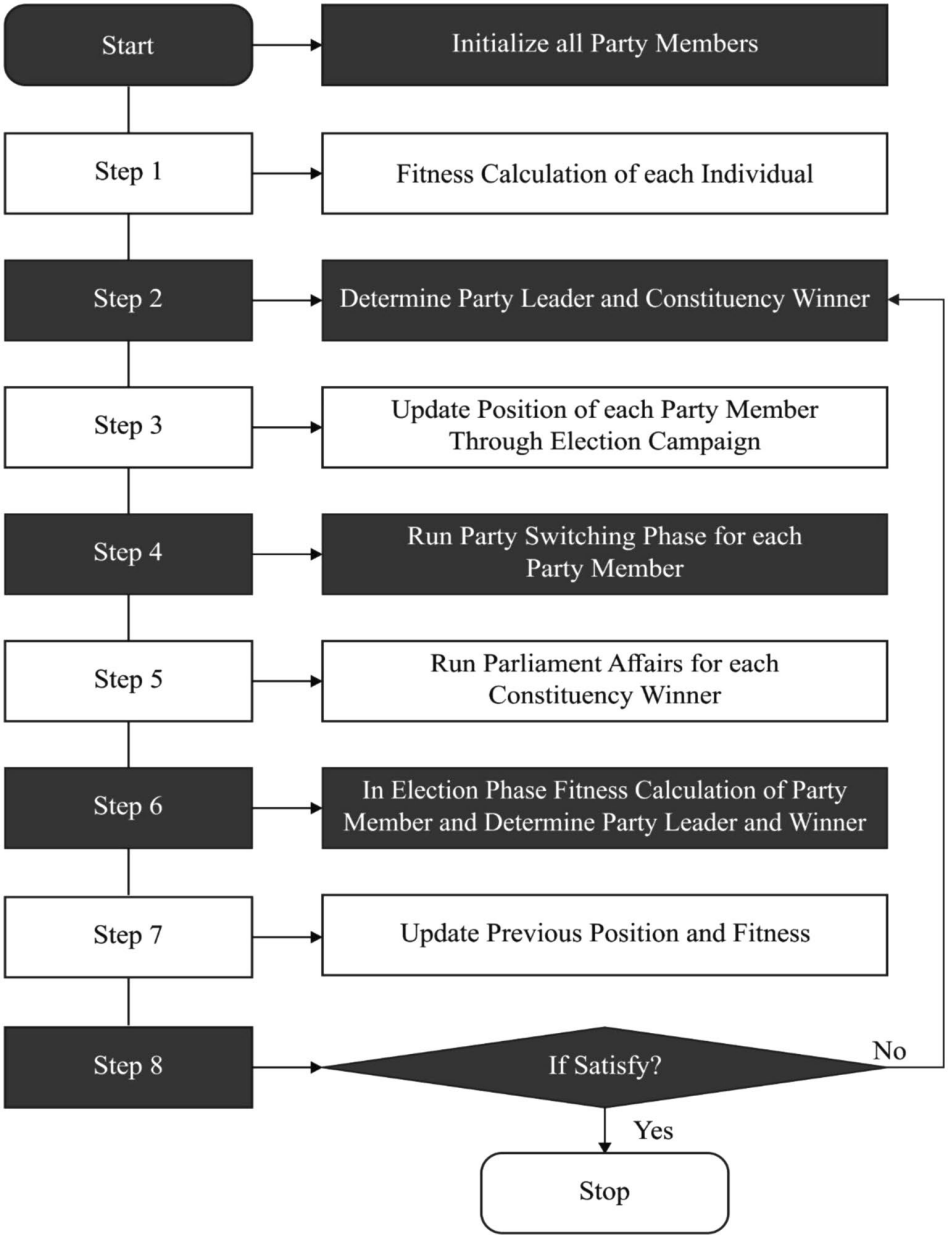
Flowchart of political optimizer.

Figure 3 depicts the flowchart of PO. Initially, the input parameters are initialized. Then the fitness function is calculated for all the individual. For these individuals the party leader and constituency winner is defined, where the position of party leader is updated through election campaign. Afterward through Parliamentary Affairs the party leader is defined and constituency winner, all the parliamentarians aiming to enhance their performances by mimicking the cooperation. In Election stage's it aim is to calculate the fitness of each candidate contest in constituency and update the previous position and fitness which results in performance improvement.

### Experimental validation

This section analyses the OERNN-BPP technique's findings using annual biofuel production data that was gathered from China between January 2015 and June 2020 (https://apps.fas.usda.gov/newgainapi/api/Report/DownloadReportByFileName?fileName=Biofuels%20Annual_Beijing_China%20-%20People%27s%20Republic%20of_CH2022-0089.pdf). The samples in the dataset are split into training data (80%) and testing data (20%), respectively. The OERNN-BPP technique's outcomes are analysed in a variety of dimensions.

### Root-mean-square error (RMSE)

The root-mean-square deviation (RMSD) or root-mean-square error (RMSE) is a frequently used measure of the differences between values (sample or population values) predicted by a model or an estimator and the values observed. The RMSD represents the square root of the second sample moment of the differences between predicted values and observed values or the quadratic mean of these differences.

9 $$RMSE=\sqrt{\frac{\sum_{i=1}^{N}{({x}_{i}-{\widehat{x}}_{i})}^{2}}{N}}$$

RMSE = root-mean-square deviation, i = variable i, N = number of non-missing data points, ${x}_{i}$= actual observations time series, ${\widehat{x}}_{i}$= estimated time series.

### Mean absolute percentage error (MAPE)

Mean absolute percentage error (MAPE) is a metric that defines the accuracy of a forecasting method. It represents the average of the absolute percentage errors of each entry in a dataset to calculate how accurate the forecasted quantities were in comparison with the actual quantities.

Table 1 shows the OERNN-BPP Model parameter information.

**Table 1:** OERNN-BPP model parameter information.

Model	Meaning	Value
OERNN-BPP	Number of input layer nodes	8
	Number of hidden layer nodes	15
	Number of output layer nodes	1
	Epochs of training	1000
	Learning rate	0–1
	Momentum coefficient	0–1
	Batch Size	100

**Figure 4:**
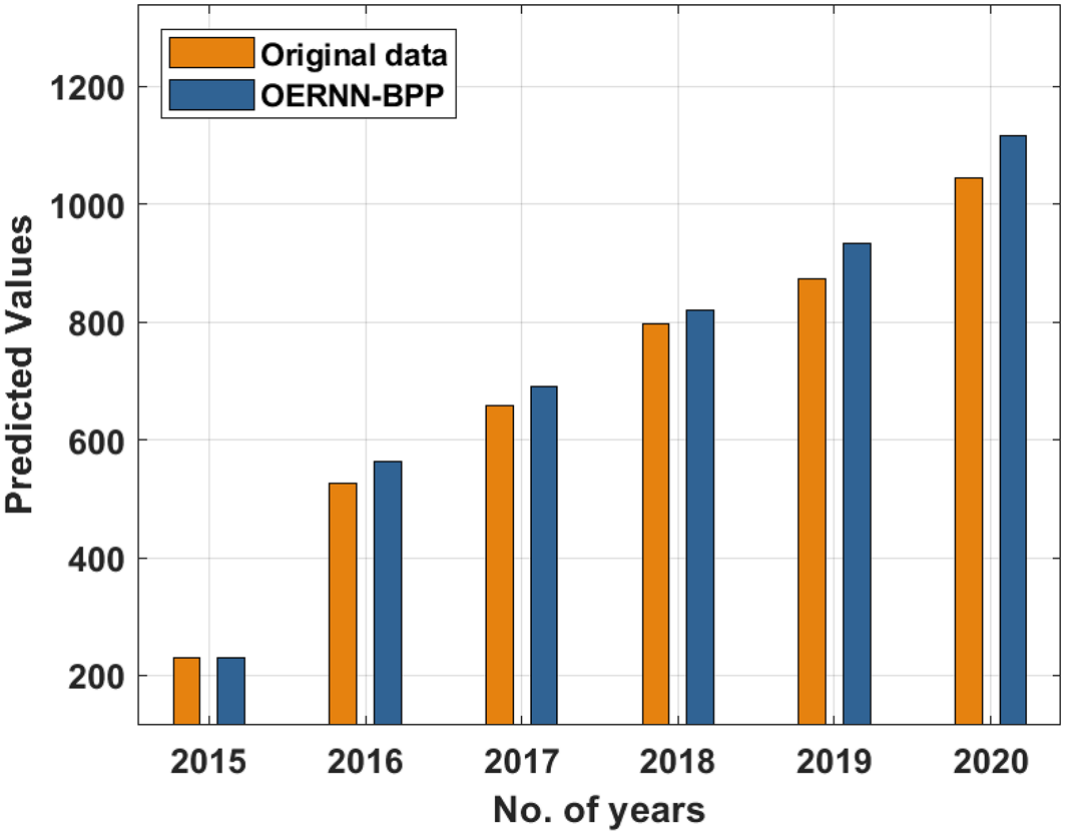
Result analysis of OERNN-BPP model.

Firstly, a brief investigation of the biofuel production rate of the OERNN-BPP model is investigated for a period of 6 years (2015–200) in Fig. 4 and Table 2. The results demonstrated the original data, predicted data by OERNN-BPP technique, and divergence rate. The obtained values portrayed that the OERNN-BPP technique has effectively predicted the biofuel production rate and the divergence rate is found to be minimal. Moreover, the divergence at a gets increased with the increase in duration.

**Table 2:** Results analysis of proposed OERNN-BPP model.

No. of years	Original data	OERNN-BPP	Divergence data
2015	231	231	0
2016	527	562.43	35.43
2017	658	691.02	33.02
2018	797	819.33	22.33
2019	874	932.75	58.75
2020	1045	1116.23	71.23

**Figure 5:**
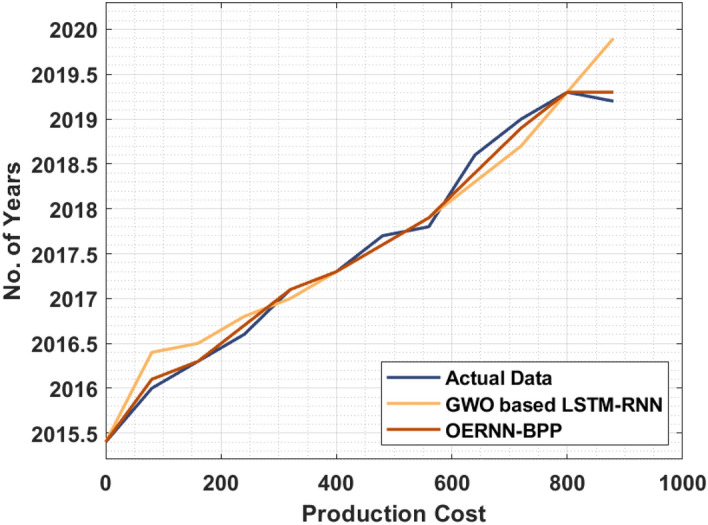
Results of biofuel production.

Another results analysis of the OERNN-BPP technique takes place interms of biofuel production cost for certain duration in Fig. 5. The figure portrayed that the OERNN-BPP technique has depicted effective performance with as light difference in the actual and predicted data. At the same time, the GWO based LSTM-RNN model has tried to demonstrate reasonable outcomes. However, the OERNN-BPP technique has out performed the existing one with a higher predictive outcome.

The existing approaches are empirical mode decomposition (EMD)- (Glow worm Swarm Optimization (GSO)-Long Short-Term Memory (LSTM)—Recurrent Neural Networks (RNNs))- called as EMD-(GWO-LSTM-RNN)-ADD, empirical mode decomposition (EMD)- (Glow worm Swarm Optimization (GSO)-Long Short-Term Memory (LSTM)—Recurrent Neural Networks (RNNs) + adaptive wavelet neural network (AWNN))- called as EMD-(GWO-LSTM-RNN + AWNN)-ADD, and empirical mode decomposition (EMD)- adaptive wavelet neural network (AWNN))-ADD is called as EMD-AWNN-ADD.

**Figure 6:**
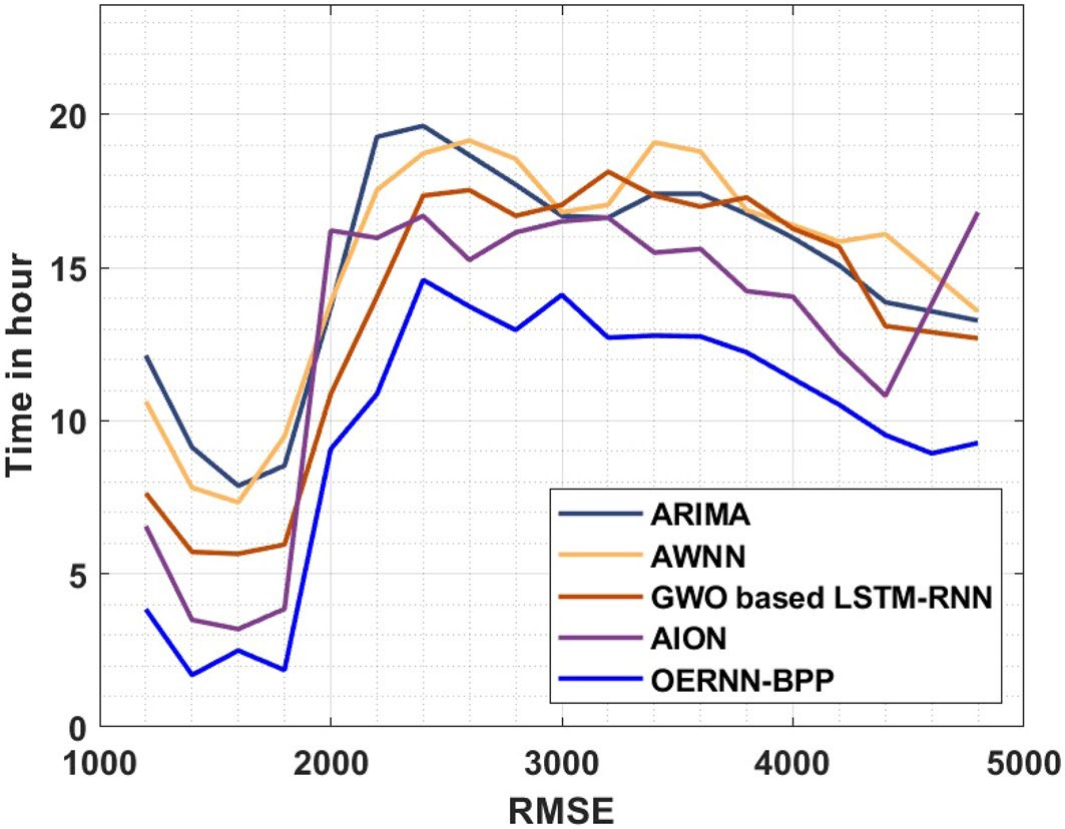
RMSE of different methods.

A brief comparative root-mean-squared error (RMSE) analysis of the OERNN-BPP technique under varying time duration takes place in Table 3 and Fig. 6. The value of RMSE tends to be minimal for better prediction outcomes. The figure reported that the AWNN technique has appeared as the poor performer with the higher RMSE values. Followed by, the ARIMA model has gained slightly enhanced RMSE value over the AWNN technique whereas the GWO based LSTM-RNN technique has demonstrated moderately RMSE value. In line with, the AION technique has exhibited reasonably reduced RMSE value. However, the proposed OERNN-BPP technique is found to be efficient with the minimum RMSE values under varying time duration.

**Table 3:** RMSE analysis of OERNN-BPP model with existing techniques.

RMSE	Timein hour				
	ARIMA	AWNN	GWO based LSTM-RNN	AION	OERNN-BPP
1200	12.13438	10.63465	7.635187	6.555381	3.845477
1400	9.134918	7.815155	5.715531	3.495929	1.696047
1600	7.875144	7.335241	5.655542	3.195983	2.496080
1800	8.535026	9.494854	5.955488	3.855865	1.856026
2000	13.69410	13.87407	10.87461	16.21365	9.074907
2200	19.27310	17.53341	14.05404	15.97369	10.87423
2400	19.63304	18.73320	17.35344	16.69356	14.60392
2600	18.67321	19.15312	17.53341	15.25382	13.73389
2800	17.71338	18.55323	16.69356	16.15366	12.96385
3000	16.69356	16.81354	17.05350	16.51360	14.11400
3200	16.63357	17.05350	18.13331	16.63357	12.71390
3400	17.41343	19.09313	17.35344	15.49378	12.78389
3600	17.41343	18.79319	16.99351	15.61376	12.75389
3800	16.75355	16.87353	17.29346	14.23400	12.23398
4000	15.97369	16.39362	16.27364	14.05404	11.37412
4200	15.07385	15.85371	15.67375	12.25436	10.52428
4400	13.87407	16.09367	13.09421	10.81462	9.534470
4600	13.57407	14.83390	12.89421	13.81408	8.934570
4800	13.27407	13.57412	12.69421	16.81354	9.274680

**Figure 7:**
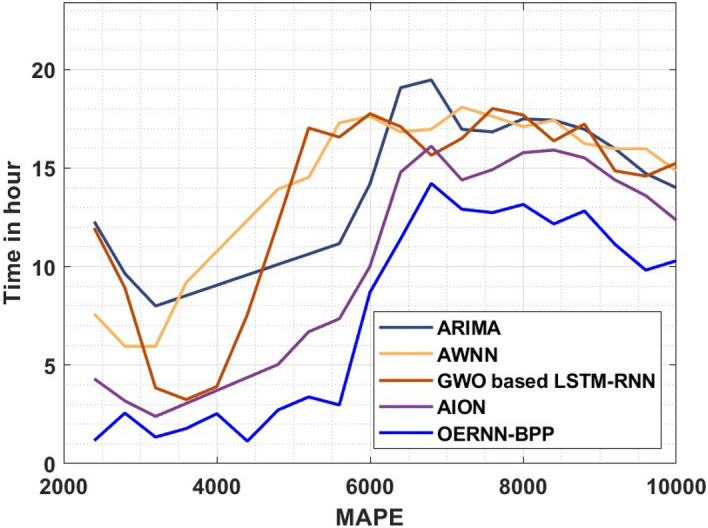
MAPE of different methods.

A detailed comparative MAPE analysis of the OERNN-BPP approach under varying time duration take place in Table 4 and Fig. 7. The value of MAPE tends to be lower for better prediction result. The figure stated that the AWNN manner has demonstrated as the least performer with the maximum MAPE values. Besides, the ARIMA approach has attained some what improved MAPE value over the AWNN technique where as the GWO based LSTM-RNN technique has demonstrated moderately MAPE value. Similarly, the AION method has exhibited reasonably lower MAPE value. However, the proposed OERNN-BPP methodology is established to effectual with the decreased MAPE value sunder varying time duration.

**Table 4:** MAPE analysis of OERNN-BPP model with existing techniques.

MAPE	Timein hour				
	ARIMA	AWNN	GWO based LSTM-RNN	AION	OERNN-BPP
2400	12.2839	7.602508	11.95422	4.305756	1.166730
2800	9.646495	5.954132	8.921209	3.184860	2.563964
3200	7.998119	5.954132	3.844211	2.393639	1.346484
3600	8.525599	9.184950	3.250795	3.052990	1.780101
4000	9.053080	10.76739	3.910146	3.712340	2.533315
4400	9.580560	12.34983	7.569496	4.371691	1.136730
4800	10.10804	13.93227	12.22885	5.031041	2.720146
5200	10.63552	14.52569	17.03122	6.690392	3.383561
5600	11.16300	17.29496	16.56968	7.349742	2.976976
6000	14.19601	17.62464	17.75651	10.00909	8.695274
6400	19.07521	16.83342	17.09716	14.78943	11.40260
6800	19.47082	16.96529	15.64658	16.10813	14.21788
7200	16.96529	18.08618	16.50374	14.39382	12.90918
7600	16.83342	17.62464	18.02025	14.92130	12.73634
8000	17.49277	17.09716	17.69057	15.77845	13.15195
8400	17.42683	17.42683	16.37187	15.91032	12.16195
8800	16.96529	16.24000	17.22903	15.51471	12.82227
9200	15.97626	15.97626	14.85536	14.39382	11.12292
9600	14.72349	15.97626	14.59162	13.60260	9.820160
10,000	13.99821	14.85536	15.25097	12.34983	10.29674

**Figure 8:**
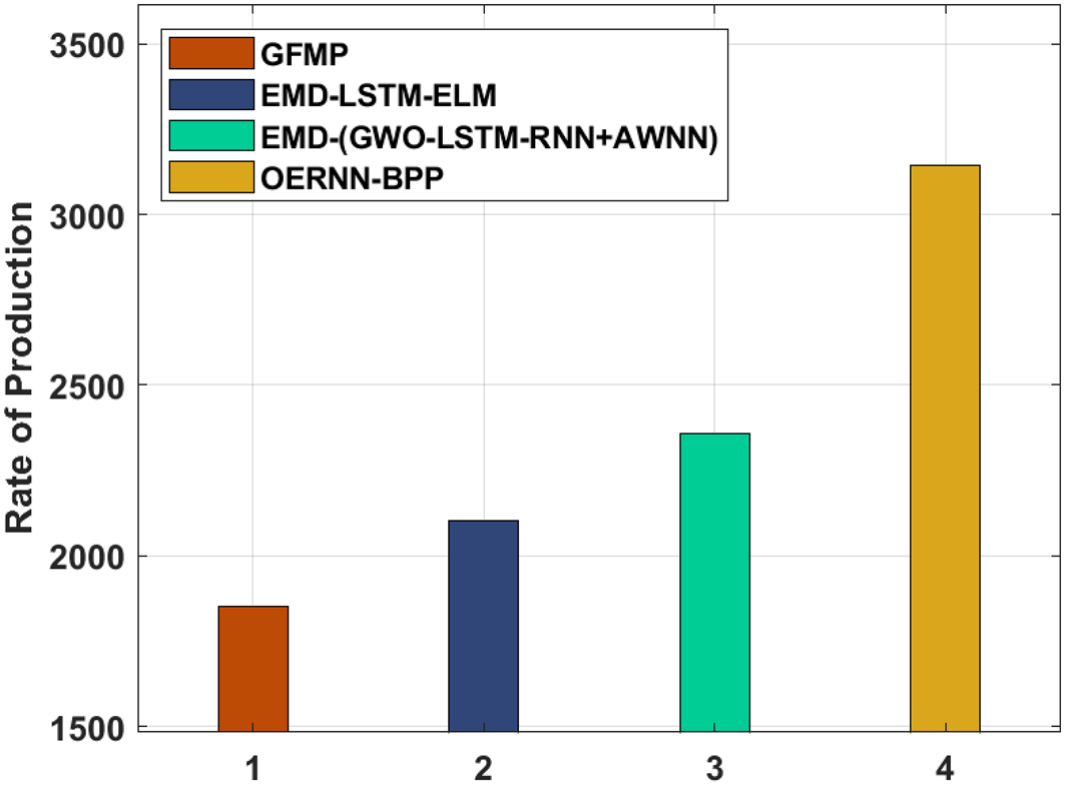
Rate of production on various methods.

Finally, a CT analysis of the OERNN-BPP technique with existing techniques is made in Table 5 and Fig. 8. The experimental results highlighted that the GFMP technique has offered worse outcomes with the least CT of 1853.391. Besides, the EMD-LSTM-ELM and EMD-(GWO-LSTM-RNN + AWNN) techniques have tried to show moderate outcomes with the CT of2101.430 and 2356.358 respectively. However, the proposed OERNN-BPP technique has resulted in superior outcomes with a maximum CT of 3145.152. From the detailed result analysis, it is ensured that the OERNN-BPP technique is found to be an effective tool to predict bio fuel productivity.

**Table 5:** Rate of production on various methods.

Methods	Rate of production
GFMP	1853.391
EMD-LSTM-ELM	2101.430
EMD-(GWO-LSTM-RNN + AWNN)	2356.358
OERNN-BPP	3145.152

## Conclusion

In this study, a new OERNN-BPP technique has been presented to predict the productivity of bio fuels. The OERNN-BPP technique encompasses four major processors namely EMD based processing, FTC based reconstruction, ERNN based prediction, and PO based hyper parameter optimization. Once the input data is pre-processed and reconstructed, the actual prediction process is carried out by the use of ERNN model. Moreover, the hyper parameters of the ERNN model namely learning rate, batch size, momentum, and weight decay are optimally adjusted by the use of PO and thereby raise the predictive outcome to a maximum extent. A thorough simulation analysis is conducted in order to show how the OERNN-BPP technique has improved, and the findings are examined from several angles. The testing results showed that the OERNN-BPP technique outperformed recently published predictive models of biofuel production. It shows that, the EMD-LSTM-ELM and EMD-(GWO-LSTM-RNN + AWNN) techniques have tried to show moderate outcomes with the CT of2101.430 and 2356.358 respectively. However, the proposed OERNN-BPP technique has resulted in superior outcomes with a maximum CT of 3145.152. Advanced hybrid deep learning architectures may be presented in the future to improve the predicted outcome.

## Data Availability

The datasets used and analyzed during the current study are available from the corresponding author on request.
